# Pleomorphic Adenoma of the Palate: A Case Report

**DOI:** 10.7759/cureus.4308

**Published:** 2019-03-25

**Authors:** Praszanth Arumugam, Pradeep J Christopher, Senthil Kumar, Srivatsa Kengasubbiah, Vandana Shenoy

**Affiliations:** 1 Oral and Maxillofacial Surgery, Thai Moogambigai Dental College & Hospital, Chennai, IND

**Keywords:** pleomorphic adenoma., minor salivary gland tumor, palate

## Abstract

Pleomorphic adenoma is the most common benign tumor affecting the major salivary glands and infrequently arises from the minor salivary glands. It involves the parotid of the major salivary glands and the palate of the minor salivary glands. This article presents a case of pleomorphic adenoma of the palatal minor salivary gland treated successfully by surgical excision.

## Introduction

Pleomorphic adenoma (PA) is a benign mixed tumor composed of epithelial and myoepithelial cells arranged in various morphological patterns, demarcated from the surrounding tissues by a fibrous capsule. PA is a salivary gland tumor that affects both the major and minor salivary glands and accounts for 40% to 70% of all tumors [1]. The parotid gland is the most commonly affected major salivary gland. Globally, 13.9% to 51.4% of all salivary gland tumors arise from an intraoral site, and 34.7% to 67.1% of them are benign [2]. PA occurs in the fourth, fifth, and sixth decades of life and is found more commonly in women (60%) than in men (40%) [3]. Among intraoral salivary glands, PA affects the palate most commonly (42.63%), followed by the lip (10%), buccal mucosa (5.5%), retromolar area (0.7%), and the ﬂoor of the mouth [4].

## Case presentation

A 38-year-old male patient reported to the department of oral and maxillofacial surgery at Thai Moogambigai Dental College and Hospital in Chennai, Tamil Nadu, India. The patient’s chief concern was swelling in his upper left back tooth region. History revealed the swelling was painless and gradually grew over one year to its present size. There were no other symptoms (e.g., numbness, dysphagia, stridor, speech, or masticatory difficulties) due to the lesions. There was no history of trauma, fever, or similar swelling elsewhere in the body. Past medical history revealed the patient was healthy and had no systemic diseases nor deleterious habits. Past dental history revealed extraction of 25 two years prior to presentation.

On general physical examination, the patient was moderately built and conscious, with a normal gait. His vital signs were within normal limits. The extraoral examination showed no facial asymmetry or lymphadenopathy.

**Figure 1:**
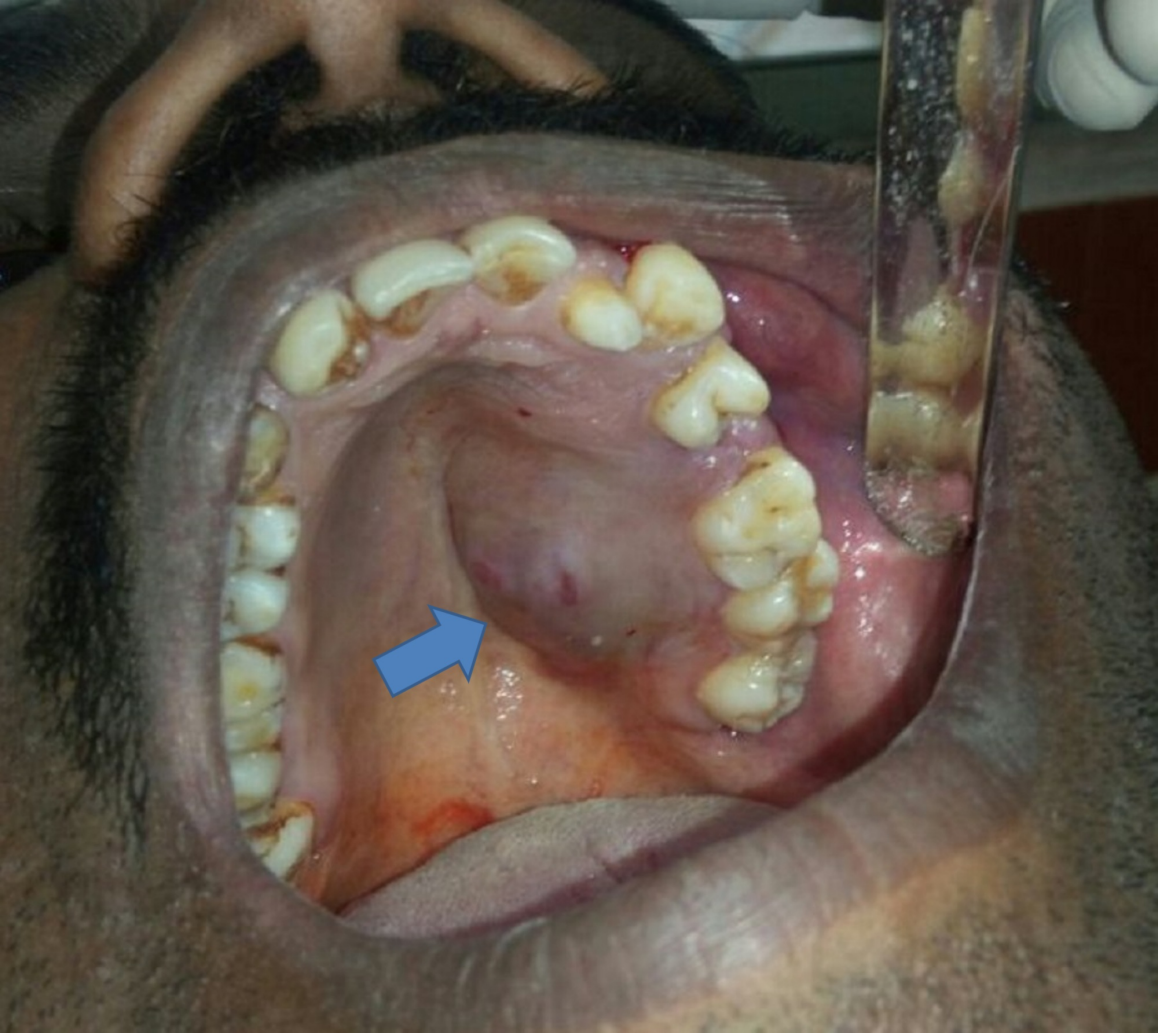
Pleomorphic adenoma, clinical view

On intraoral examination, we noted a single, ovoid-shaped swelling measuring 3 cm x 2 cm in the left posterolateral surface of the hard palate. The swelling extended anteriorly from the region of 23 to the region of 27, posteriorly. Medially, it extended from the midline of the hard palate and distal aspect of the region of 27 laterally (Figure 1). The overlying mucosa appeared healthy and smooth with no secondary changes. On palpation, the swelling was unilocular, nontender, nonpulsatile, firm, and immovable with well-defined margins. The mucosa over the lesion was stretched and nonpinchable.

The results of the patient’s routine blood investigations were within normal limits. Intraoral hard tissue examination revealed no anomalies of the teeth in relation to the lesion. The orthopantomogram did not reveal pathological changes in the bone structures. Due to the clinical examination, outlook, and history of the lesion, we decided to surgically excise the lesion with local anesthesia.

**Figure 2:**
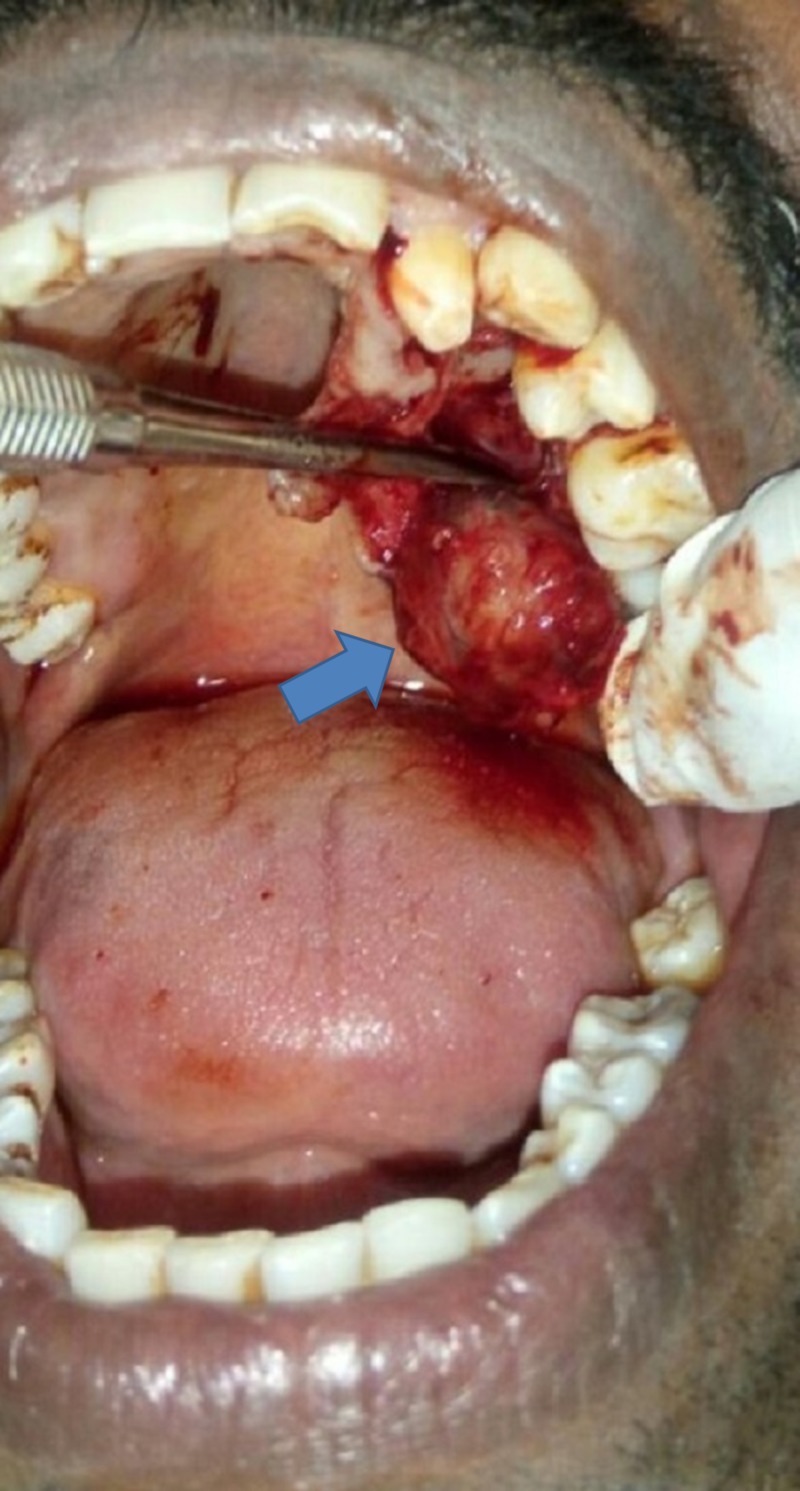
Pleomorphic adenoma, intraoperative view

**Figure 3:**
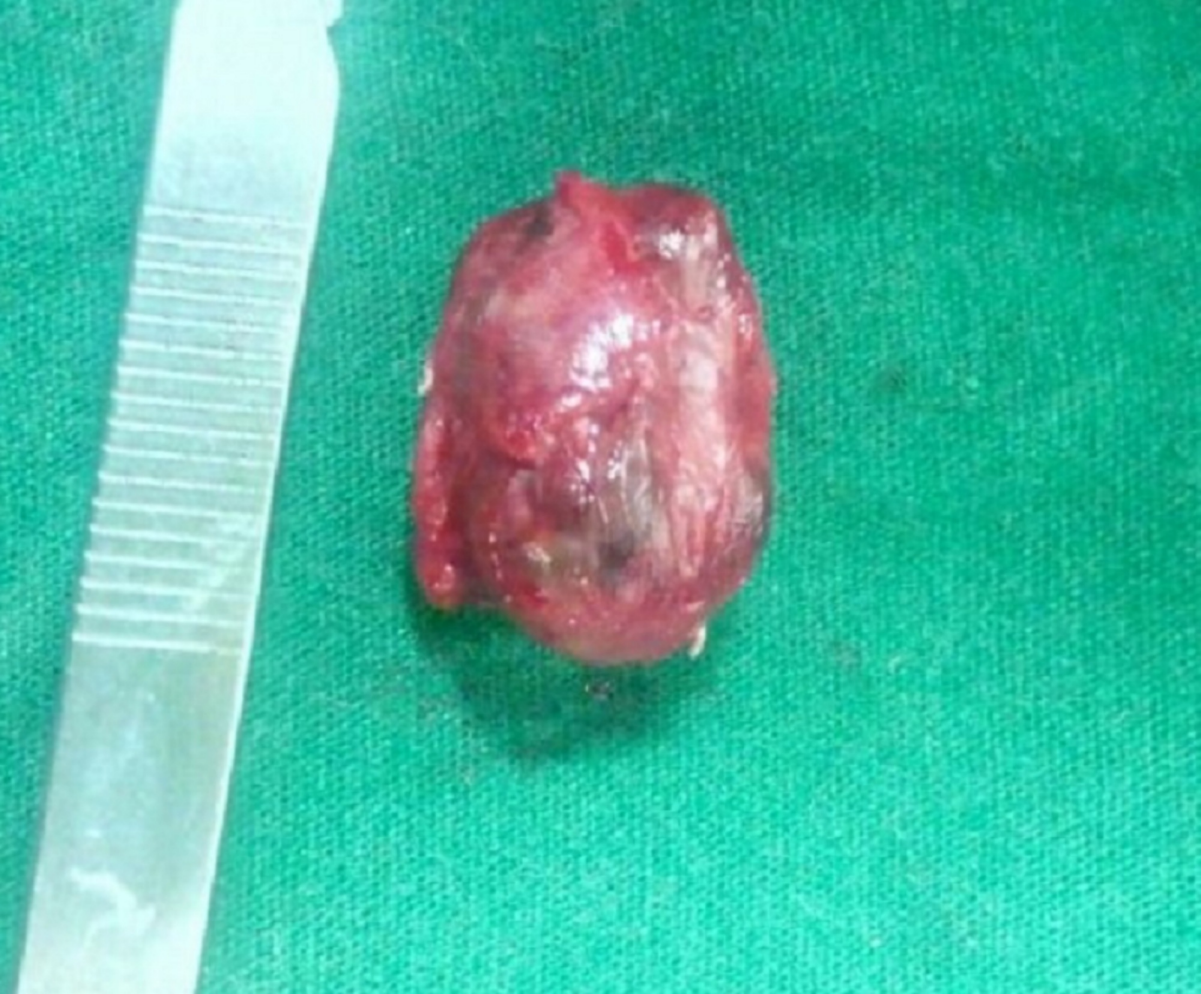
Pleomorphic adenoma, excised

A crevicular incision was made from mesial papilla of 22 to the distal papilla of the region of 27 using a No. 15 Amkay Surgical Bard-Parker® blade (Amkay Products Pvt. Ltd., Maharashtra, India) (Figure 2). The mucoperiosteal flap was reflected, and the whole encapsulated tumor mass was excised along with the mucoperiosteum and the eroded bone of the palate with the boundary line localized in the surrounded healthy tissue (Figure 3). Hemostasis was achieved and wound closure done using 3-0 silk.

**Figure 4:**
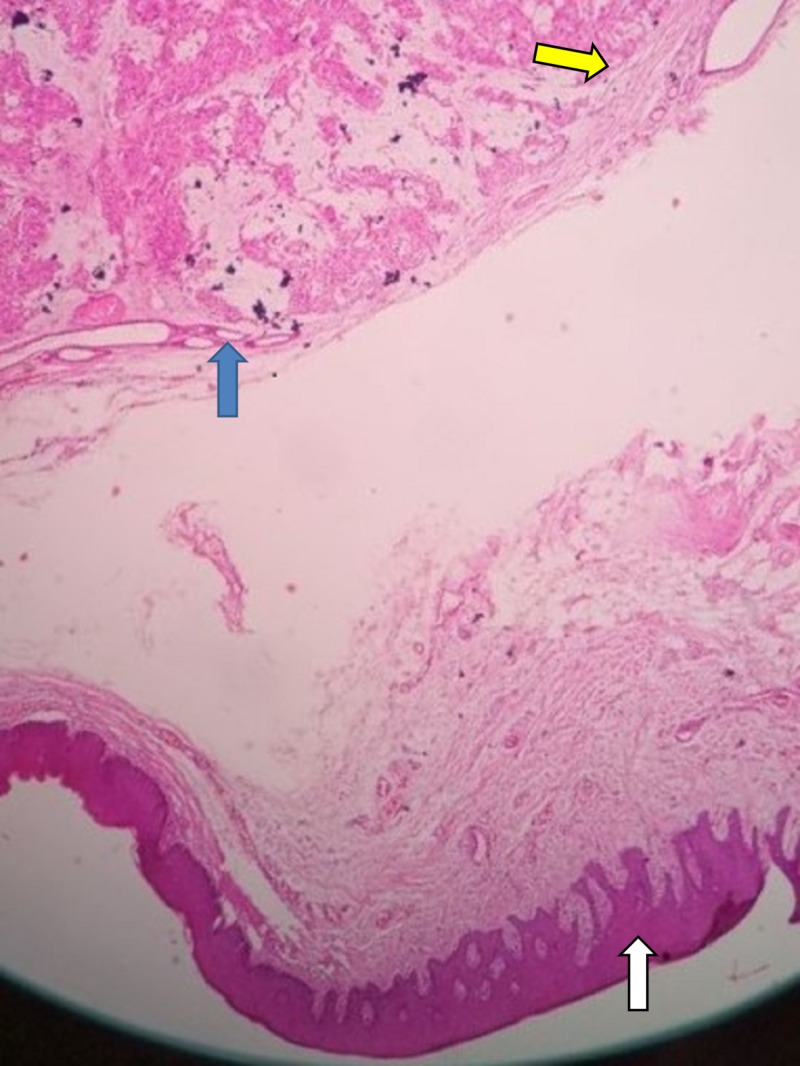
Pleomorphic adenoma, histopathological examination. Blue arrow denotes islands of myoepithelial cells; yellow arrow denotes mesenchymal connective tissue layers; white arrow denotes parakeratinized stratified squamous epithelium.

The histopathological examination of the mass revealed parakeratinized stratified squamous epithelium along with connective tissue. The underlying connective tissue showed a well-encapsulated mass of sheets and islands of myoepithelial cells and very few duct-like spaces filled with eosinophilic material. Islands of myoepithelial cells were surrounded by eosinophilic myxoid material (Figure 4). This confirmed the diagnosis of PA. The postoperative period was uneventful. The patient is under regular follow-up, and there is no evidence of recurrence after six months of follow-up.

## Discussion

According to Vuppalapati, PA derives its name from the architectural pleomorphism visible on light microscopy. PA is also known as mixed tumor-salivary gland type, a phrase that describes its pleomorphic appearance as opposed to its dual origin from epithelial and myoepithelial elements. The “mixed tumor” aspect accounts for 73% of all salivary gland tumors. The palate is the most common site for PA and corresponds to small glands [3].

Spiro et al. conducted a study of 2078 patients with salivary gland neoplasia and reported that 20% to 40% of all salivary gland tumors arise from minor salivary glands. The mixed minor salivary tumors affect mostly patients in their fourth to sixth decades of life. While it can affect both sexes, the condition has a slight predilection for women over men [4].

PA arises in the oral cavity as a painless, slow-growing, firm swelling commonly seen on the posterior lateral aspect of the palate and presents as a smooth, dome-shaped mass [5]. Because of the tightly bound nature of the hard palate mucosa, it appears to be fixed. PA tumors in the lips and buccal mucosa are freely movable. PA of the palate is seldom allowed to attain a size greater than 1 cm to 2 cm in diameter because it causes difficulty in mastication, speech, and swallowing [6]. PA is detected and treated earlier than tumors of the major salivary glands. If the overlying mucosa is ulcerated, and the ulceration is not due to any trauma or biopsy, malignancy should be suspected [1].

Histologically, the tumor consists of epithelial, myoepithelial, and mesenchymal components arranged in a complex pattern. The epithelial cells arranged in sheets and nests of cells give rise to glandular, ductal structures filled with an eosinophilic coagulum. Squamous metaplasia and keratin pearls are also present. Myoepithelial cells are a distinct feature of PA. Plasmacytoid myoepithelial cells are an exclusive feature of minor salivary gland tumors. Spindle cells, clear cells, and oxyphilic cells may also be present. The mesenchymal component gives rise to chondroid, myxoid, and osseous areas. The salivary gland parenchyma undergoes fibrosis, giving rise to a false capsule [5].

Daryani et al. reported a case of PA of the palate and made the following differential diagnosis: hematoma (bluish discoloration), mucocele, necrotizing sialometaplasia, mucoepidermoid carcinoma, adenoid cystic carcinoma, and polymorphous low-grade adenocarcinoma [5]. Sharma et al. also reported a similar swelling where the differential diagnosis was a neuroma, palatal abscess, and neurofibroma [1].

The treatment of PA is essentially surgical excision [7-8]. Because these tumors are radioresistant, radiation therapy is contraindicated [9]. Though these benign tumors are well-encapsulated, resection of the tumor with an adequate margin of grossly normal surrounding tissue is necessary to prevent local recurrence because these tumors are known to have microscopic pseudopod-like extensions into the surrounding tissue due to "dehiscence" in the capsule [10]. The recurrence of PA is attributed to implantation from capsule rupture, islands of tumor tissue left behind after surgery, and the multicentric nature of PA. Therefore, long-term follow-up is required [11].

## Conclusions

PA of the palate is a very rare entity, usually seen in adult patients. The most common symptom is a slow-growing, painless submucosal mass on the hard palate. Definitive diagnosis lies in the histopathological examination, and treatment is by surgical excision with wide margins. Excellent results are seen if the wound is allowed to granulate and heal by itself. Recurrences are uncommon but may be seen on long-term follow-up.
